# Segmental testicular infarction: A case report

**DOI:** 10.3892/etm.2014.2151

**Published:** 2014-12-19

**Authors:** YUE-HONG SHEN, YI-WEI LIN, XUN-WEN ZHU, BO-SEN CAI, JUN LI, XIANG-YI ZHENG

**Affiliations:** 1 Department of Urology, The First Affiliated Hospital, School of Medicine, Zhejiang University, Hangzhou, Zhejiang 310003, P.R. China; 2 Department of Ultrasonography, The First Affiliated Hospital, School of Medicine, Zhejiang University, Hangzhou, Zhejiang 310003, P.R. China; 3 Department of Pathology, The First Affiliated Hospital, School of Medicine, Zhejiang University, Hangzhou, Zhejiang 310003, P.R. China

**Keywords:** infarction, segmental, testicular

## Abstract

The incidence of segmental testicular infarction is extremely low. The condition usually presents with acute scrotal pain and may be confused clinically and radiologically with a testicular tumor or torsion. To the best of our knowledge, only a few cases have been reported in the English literature. In this study, we present a case of segmental testis infarction in a 23-year-old male with an acute onset of testicular pain. The diagnosis of testicular infarction was considered following sonography examination. Hemorrhagic infarction of the testis was confirmed by surgical exploration and pathological examination. Partial orchiectomy was performed. Although it is uncommon, segmental testicular infarction should be taken into consideration when acute scrotal pain is encountered, since the therapeutic strategy could be conservative.

## Introduction

Global infarction of the testes is a common diagnosis in urologic emergencies. Possible causes include torsion of the spermatic cord, incarcerated hernia, severe epididymitis and iatrogenic injury (1). In contrast, the segmental infarct of the testicle is uncommon. The condition is typically idiopathic and usually affects patients between the second and the fourth decades of life. The classical clinical presentation of segmental testicular infarction would be acute onset of scrotal pain which mimics testicular torsion. However the radiological presentation of segmental testicular infarction would resemble that of testicular tumor, and tends to prompt treatment by radical surgery. Thus, the diagnosis was usually established following orchidectomy (2) Only a few cases of segmental testicular infarction have been reported previously (3,4). The present study describes a case of acute scrotum in a 23-year-old male.

## Case report

**Figure 1:**
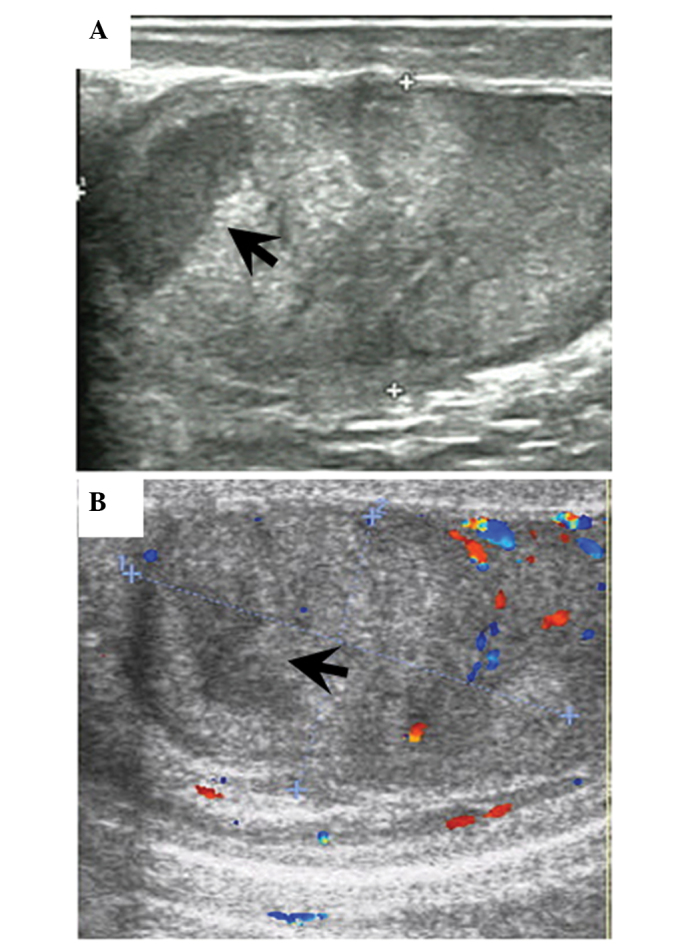
(A) Scrotal sonography demonstrated a wedge-shaped hypoechoic lesion located in the upper pole of the testis (arrow). (B) Color Doppler sonography revealed that the hypoechoic lesion was completely flowless (arrow).

A 23-year-old male with unremarkable past medical history was admitted due to worsening right testicular pain that had been present for three days. Physical examination revealed a mildly swollen right testis with tenderness in the upper pole. The testis was otherwise normal with no mass or hernia. Results of a complete blood count, urinalysis and tumor marker profile were all within the normal range. Color Doppler sonography demonstrated a flowless, well-demarcated, hypoechoic mass located in the upper pole of the right testis (Fig. 1). The remaining area of the testis exhibited normal echogenicity and vascularity. Since the underlying cause of the testicular pain was unclear, surgical exploration was performed.

**Figure 2:**
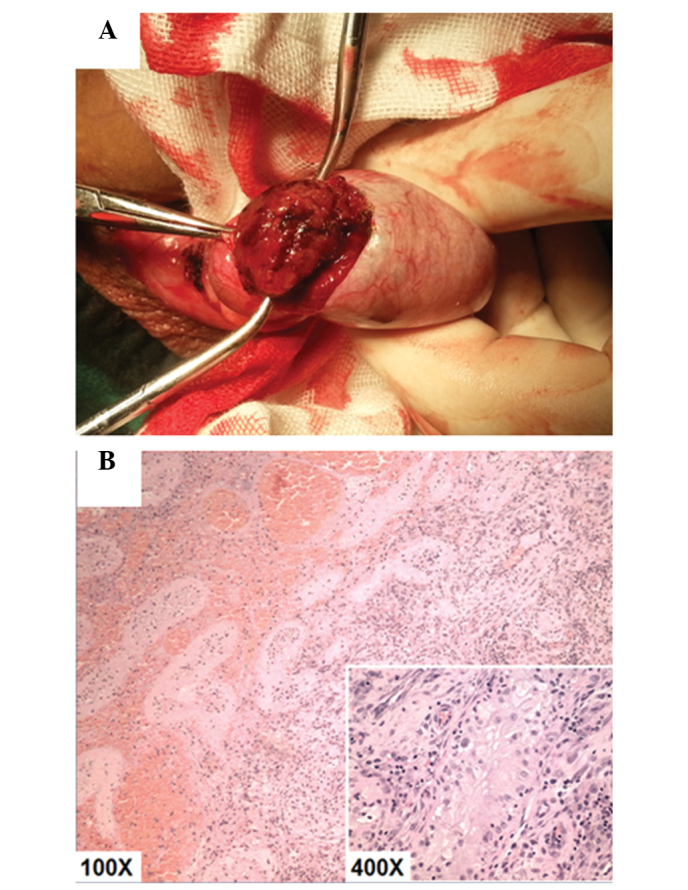
(A) Intraoperative photograph showing the hemorrhagic necrosis of the upper pole (dark appearance). (B) The hematoxylin and eosin section showed diffuse interstitial hemorrhage (magnification, ×100), and the surrounding seminiferous tubule lined only with sertoli cells (magnification, ×400).

The patient’s scrotum was immediately explored via a scrotal approach. During the surgery, the upper pole of the testis showed a faint blue discoloration; however, no torsion of the spermatic cord or the epididymis was detected. The tunica albuginea of the testicle was incised, revealing necrosis of the upper pole with grossly normal residual testis tissue (Fig. 2A). Partial orchiectomy was performed. The pathological examination of the excised specimen showed diffuse hemorrhagic infarction of the testis tissue, with the surrounding normal tissue exhibiting the characteristic histological features of sertoli cell-only syndrome (Fig. 2B). The surgery was successful, with no complications, and the patient fully recovered. Written informed consent was obtained from the patient prior to publication of this case report and of any accompanying images.

## Discussion

Segmental infarct of testicle is a rare clinical entity that is usually diagnosed following orchiectomy. Since the first reported case in 1909 (5), <70 cases have been reported. The condition typically has an idiopathic etiology, although in certain cases predisposing factors for segmental infarction have been noted, such as hypercoagulability disorders, vasculitis, trauma, infection, torsion and iatrogenic vascular injury (3–8). In the present case, the presence of sertoli cell-only syndrome would possibly indicate an association between spermatogenesis and segmental testicular infarction, as similar spermatogenesis arrest morphology was described in the case reported by Baratelli *et al* (9); however, whether there exists a convincing association warrants further study.

The most common symptom of segmental testicular infarction is testis pain, which is unspecific and indistinguishable from that of other scrotal diseases. Differential diagnosis should thus depend on laboratory tumor marker screening and imaging. Although a previous study proposed the diagnostic value of magnetic resonance imaging in segmental testicular infarction (10), scrotal ultrasound remains the most simple and useful tool to distinguish segmental testicular infarction from other diseases (11). The use of imaging to differentiate segmental testicular infarction from testis tumor relies on the recognition that the typical ischemic region should resemble the lobular morphology of the testicle (12); therefore, the characteristic ultrasound finding would be an avascular, wedge-shaped, hypoechoic lesion with well-defined borders. Additional shear-wave elastography would increase the accuracy of the diagnosis (13).

The management strategy for segmental testicular infarction remains controversial. The primary intention of surgical intervention is the salvage of testicular tissue along with the pathological exclusion of malignancy. Testis-sparing surgery is therefore an optimal surgical choice, particularly for younger patients (14). Despite this, the majority of segmental testicular infarction cases in the literature have resulted in radical orchiectomy (1,2,4,7), since it is difficult to establish a firm diagnosis preoperatively, and the possibility of testis tumor should always be taken into consideration in these cases. A conservative strategy is also recommended. In a case series by Madaan *et al* (15), 16 out of 19 cases of segmental testicular infarction were successfully managed by careful observation, and nine patients had gradual regression of the lesion in the follow-up ultrasonography. Conservative management with careful observation is also considered feasible and safe whenever segmental testicular infarction can be diagnosed with certainty.

In conclusion, we propose that segmental testicular infarction should be considered in patients with acute testicular pain. Either surgical exploration or careful observation is suitable when a preoperative laboratory and imaging evaluation supports a firm diagnosis.
